# Efficacy and safety of precision-guided transjugular extrahepatic portosystemic shunt (TEPS) in the management of cavernous transformation of the portal vein with portal hypertension: a case series

**DOI:** 10.1007/s12072-024-10656-8

**Published:** 2024-04-09

**Authors:** Liu Zhang, Yi-Jiang Zhu, Xue-qing Wang, Rui-feng Wang, Li Dong, Liang Yin, Wei-Fu Lv, De-Lei Cheng, Chun-Ze Zhou

**Affiliations:** 1 Interventional Radiology Department, The First Affiliated Hospital of USTC, Division of Life Sciences and Medicine, University of Science and Technology of China, No. 17 Lujiang Road, Luyang District, Hefei, 230001 Anhui People’s Republic of China; 2 Nephrology Department, The Second Affiliated Hospital of Anhui Medical University, Hefei, 230001 Anhui People’s Republic of China; 3 Bengbu Medical University, Bengbu, 233030 Anhui People’s Republic of China

**Keywords:** Portosystemic shunt, Portal hypertension, Thrombosis, Cavernous transformation

## Abstract

**Background and aims:**

Performing a Transjugular intrahepatic portal system shunt (TIPS) in patients with portal vein cavernous transformation (CTPV) poses significant challenges. As an alternative, transjugular extrahepatic portal vein shunt (TEPS) may offer a potential solution for these patients. Nonetheless, the effectiveness and safety of TEPS remain uncertain. This case series study aimed to evaluate the efficacy and safety of TEPS in treating patients with CTPV portal hypertension complications.

**Methods:**

The study encompassed a cohort of 22 patients diagnosed with CTPV who underwent TEPS procedures. Of these, 13 patients manifested recurrent hemorrhagic episodes subsequent to conventional therapies, 8 patients grappled with recurrent or refractory ascites, and 1 patient experienced acute bleeding but refused endoscopic treatment. Comprehensive postoperative monitoring was conducted for all patients to rigorously evaluate both the technical and clinical efficacy of the intervention, as well as long-term outcomes.

**Results:**

The overall procedural success rate among the 22 patients was 95.5% (21/22).During the TEPS procedure, nine patients were guided by percutaneous splenic access, three patients were guided by percutaneous hepatic access, five patients were guided by transmesenteric vein access from the abdomen, and two patients were guided by catheter marking from the hepatic artery. Additionally, guidance for three patients was facilitated by pre-existing TIPS stents. The postoperative portal pressure gradient following TEPS demonstrated a statistically significant decrease compared to preoperative values (24.95 ± 3.19 mmHg vs. 11.48 ± 1.74 mmHg, *p* < 0.01).Although three patients encountered perioperative complications, their conditions ameliorated following symptomatic treatment, and no procedure-related fatalities occurred. During a median follow-up period of 14 months, spanning a range of 5 to 39 months, we observed four fatalities. Specifically, one death was attributed to hepatocellular carcinoma, while the remaining three were ascribed to chronic liver failure. During the follow-up period, no instances of shunt dysfunction were observed.

**Conclusions:**

Precision-guided TEPS appears to be a safe and efficacious intervention for the management of CTPV.

## Introduction

Transjugular intrahepatic portosystemic shunt (TIPS) has emerged as a crucial therapeutic option for managing portal vein thrombosis (PVT) in liver cirrhosis [1, 2]. TIPS effectively controls ascites, prevents esophageal varices rebleeding, and promotes portal vein (PV) recanalization. However, TIPS poses challenges in patients with main portal vein (MPV) occlusion or cavernous transformation of the portal vein (CTPV) and small-diameter collateral vessels in liver. The success rate is merely 68–77.8% even in specific cases employing percutaneous transhepatic or percutaneous transsplenic guidance [3, 4], with an unsatisfactory long-term patency rate. The main challenge in TIPS arises from the incapacity to open MPV or due to the insufficient capacity of small PV collaterals for long-term patency.

Surgical extrahepatic portosystemic shunts, including mesocaval and splenorenal shunts, are frequently employed for the treatment of portal hypertension. These procedures involve bypassing the occluded MPV, effectively reducing the portal vein pressure (PVP). However, there are limited reports on interventional extrahepatic portosystemic shunts, possibly owing to the complexity of the surgical technique and the potential risks of significant bleeding or visceral injury. With advances in surgical techniques, imaging equipment, and covered stents, transjugular extrahepatic portosystemic shunt (TEPS) appears to be a novel exploratory method. Li reported 20 cases of mesocaval portosystemic shunt, exhibiting a 100% success rate and good short-term patency, without severe surgical complications [5]. Another case report of three patients undergoing interventional extrahepatic shunt also revealed good safety outcomes [6].

In the aforementioned reports, mesocaval portosystemic shunt necessitates open surgery, leading to increased trauma and rendering it unsuitable for patients with SMV obstruction. Additionally, unguided needle puncture for extrahepatic shunt poses a substantial risk of bleeding. Here, we present the outcomes of a single-center case series assessing the feasibility, safety, and effectiveness of precision-guided TEPS in managing CTPV with portal hypertension.

## Materials and methods

### Study design and participants

This retrospective case series study was approved by the Ethics Committee of the First Affiliated Hospital of the University of Science and Technology, China. Before any study procedures were conducted, all patients or their guardians were informed about the available treatment options, and written informed consent was obtained for the procedures.

Over the period from March 2018 to June 2023, a total of 105 patients with CTPV were treated at our center. The majority of patients experienced relief from symptoms of portal hypertension (such as abdominal pain, bleeding, or ascites) following the administration of anticoagulants, endoscopic treatment, TIPS, or splenectomy/splenic embolization. Patients who have failed traditional treatments (including portal vein recanalization, TIPS, endoscopic treatment), experience recurrent bleeding or refractory ascites despite receiving traditional treatments, or refuse to undergo the traditional treatments, are considered eligible candidates for Transjugular Extrahepatic Portosystemic Shunt (TEPS). During this period, this study consecutively enrolled 22 eligible patients with CTPV, all of whom experienced complete PV obstruction where the obstruction in the MPV exceeded 90% or the MPV trunk was replaced by fibrous cords.

In 22 patients, 20 had concurrent cirrhosis, while 2 did not have a cirrhotic background. The diagnosis of cirrhosis was primarily established through a comprehensive evaluation using clinical, radiological, and histopathological criteria. Clinical criteria included evidence of chronic liver disease, such as prolonged jaundice, hepatomegaly, ascites, or hepatic encephalopathy (HE). Radiological criteria involved imaging studies, such as ultrasound, CT scans, or MRI, demonstrating characteristic features of cirrhosis, such as nodular liver surface, splenomegaly, or signs of portal hypertension. Histopathological confirmation was obtained through liver biopsy or surgical specimens in selected cases.

Among the 22 cases, 13 patients experienced recurrent bleeding following traditional treatment, eight had recurrent/refractory ascites, and one had acute bleeding but declined endoscopic treatment. The demographic and disease status of the included patients in this study are shown in Tables 1 and 2.

**Table 1:** Baseline characteristics of patients undergoing TEPS for CTPV

Parameter	Mean ± SD,median (IQR) or absolute (percentage)
Sex	
Male	14 (63.6%)
Female	8 (36.4%)
Etiology	
Liver cirrhosis	20 (90.9%)
Non-cirrhosis	2(9.1%)
Age (years)	55.27 ± 12.10
RBC (× 10^12^/L)	3.02 ± 0.51
Hb (g/l)	78.73 ± 17.77
WBC (× 10^9^/L)	2.86 (2.04–3.91)
PLT (× 10^9^/L)	105.00 (57.00–159.00)
ALT (U/L)	22.55 ± 9.48
AST (U/L)	30.09 ± 11.85
TBIL (μmol/L)	21.30 (15.90–32.90)
ALB (g/l)	32.37 ± 5.95
BUN(mmol/L)	6.42 ± 2.39
Cr(μmol/L)	60.30 ± 15.12
Na^+^(mmol/L)	137.86 ± 5.84
PT (s)	14.90 (13.45–16.18)
INR	1.37 (1.18–1.47)
APTT (s)	32.76 ± 6.80
CP score	7.96 ± 1.89
MELD score	9.92 ± 5.23

Data are presented as mean ± standard deviation, median (interquartile range,IQR) or *n* (%). *CP score* Child–Pugh score, *MELD* model for end-stage liver disease

**Table 2:** Pre-TEPS details of patients

No.	Sex	Age	Obstruction site			Etiology	CP score	MELD score	Clinical manifestation			Treatment history						
			PV	SMV	SV				bellyache	EGVB	Ascites	EVL/ESVD	LVP	Anticoagulation	Splenectomy	TIPS	BRTO	LT
1	M	39	+	−	−	HBV	10	15	−	+	+++	+	+	−	−	−	−	−
2	F	74	+	+	+	PBC	8	3	−	−	+++	−	+	−	−	−	−	−
3	M	48	+	−	−	HBV	11	20	−	+	−	−	−	−	−	−	+	−
4	M	39	+	−	−	HBV	6	13	−	+	+	−	−	+	−	−	−	+
5	M	43	+	+	−	HBV	5	10	−	+	−	+	−	+	−	+	−	−
6	M	58	+	+	−	BCS	11	17	−	−	+++	−	+	+	−	−	−	−
7	M	52	+	−	+	HBV	10	13	−	+	+++	+	+	+	−	−	−	−
8	M	50	+	+	N/A	HBV	7	9	−	+	−	+	−	+	+	+	−	−
9	M	39	+	−	N/A	HBV	9	11	+	+	+	+	−	+	+	+	−	−
10	F	60	+	−	N/A	Unknown	6	7	−	+	+	+	−	−	+	−	−	−
11	F	50	+	+	N/A	SOS	10	8	−	+	+++	−	−	−	+	−	−	−
12	F	62	+	−	+	PBC	7	3	−	+	+ +	+	−	−	−	−	−	−
13	F	40	+	+	+	Unknown	5	7	−	+	−	+	−	+	−	−	−	−
14	F	58	+	−	−	Unknown	6	7	−	+	−	−	−	−	−	−	−	−
15	F	49	+	+	−	PBC	8	3	−	+	−	−	−	−	−	−	−	−
16	F	52	+	−	+	PMF	7	13	−	+	−	+	−	+	−	+	−	−
17	M	69	+	−	−	HBV	10	13	−	+	+++	+	+	+	−	−	−	−
18	M	58	+	+	N/A	HBV	6	10	+	−	+	−	−	+	+	−	−	−
19	M	76	+	−	N/A	HBV	9	7	−	−	+++	−	+	−	+	+	−	−
20	M	73	+	−	+	HBV	7	12	−	+	+ +	+	−	−	−	−	−	−
21	M	53	+	−	N/A	HBV	9	7	−	+	+ +	+	−	−	+	+	−	−
22	M	74	+	−	−	HBV	8	12	−	+	+ +	+	−	−	−	+	−	−

*PV* Portal vein, *SMV* Superior mesenteric vein, *SV* Splenic vein, *N/A* not applicable or not available, *BCS* Budd Chiari syndrome, *SOS* Sinusoidal obstruction syndrome, *PBC* Primary biliary cirrhosis, *PMF* primary myelofibrosis, *CP score* Child–Pugh score, *MELD score* Model for End-stage Liver Disease, *EGVB* Esophageal and gastric varices bleeding, *EVL* Endoscopic variceal ligation, *ESVD* Endoscopic selective varices devascularization, *LVP* large-volume paracentesis, *TIPS* Transjugular intrahepatic portosystemic shunt, *BRTO* Balloon-occluded retrograde transvenous obliteration, *LT* Liver transplantation

### TEPS procedure

Patients underwent abdominal enhanced computerized tomography and 3D reconstruction of the portal venous system within 7 days before the TEPS procedure to evaluate the location and degree of PV obstruction and to perform a preliminary assessment of the surgical and guidance methods that may be used during the procedure. Additionally, preoperative CT examinations allow us to evaluate the anatomical relationship among the inferior vena cava, major biliary radicals, organs, and adjacent portal collaterals. This information aids in planning the surgical procedure and identifying potential areas of concern. The TEPS procedure was performed on all patients by the same experienced surgical team (LWF, ZCZ, CDL, and ZYJ) under either local or general anesthesia (for those undergoing transmesenteric vein shunt).

**Fig. 1:**
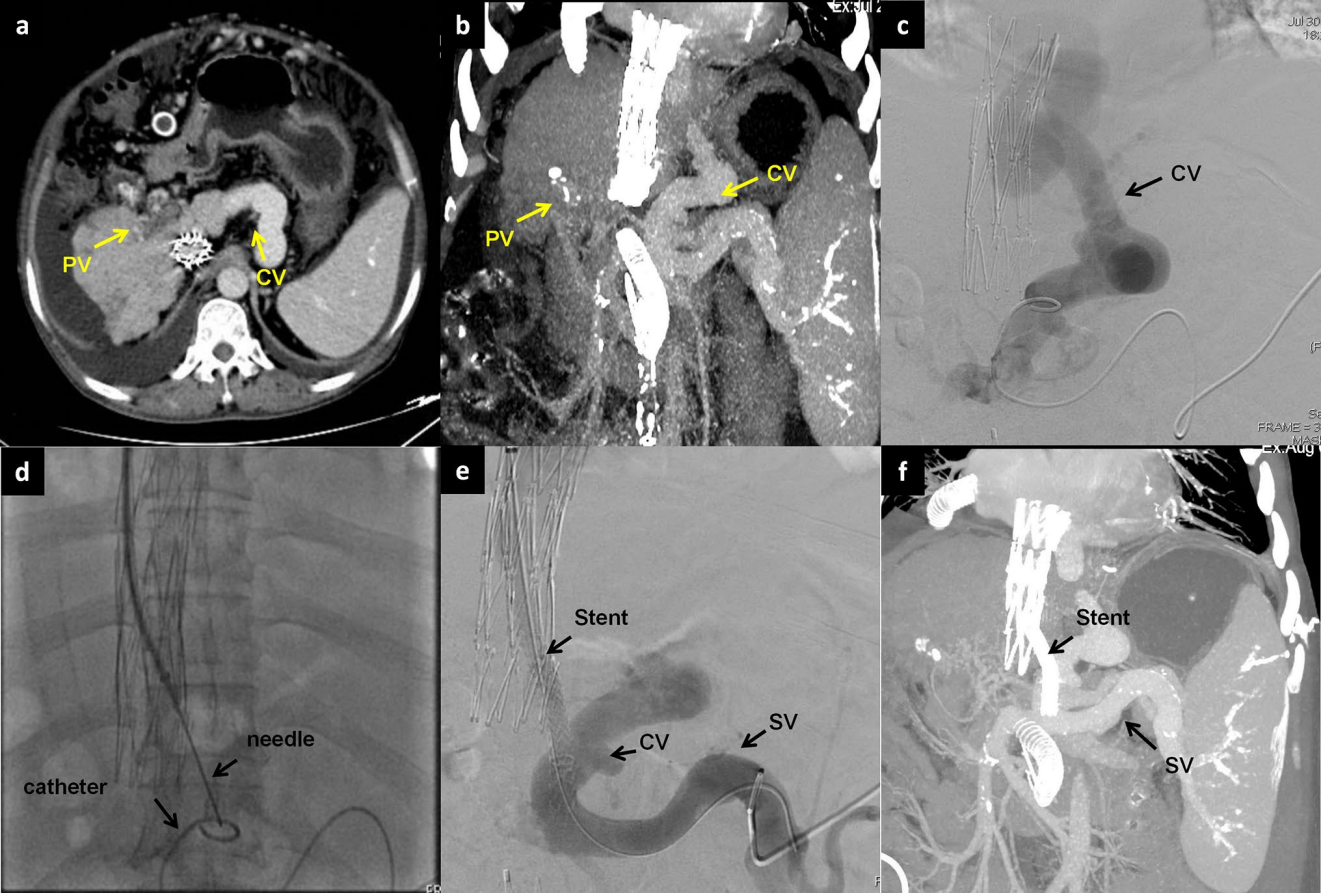
A 58-year-old male with Budd–Chiari syndrome, who had previously undergone an inferior vena cava-right atrial shunt and inferior vena cava stent implantation a decade ago, now presents with recurring episodes of esophagogastric variceal bleeding and recurrent ascites. **a**–**b** Spiral enhanced CT with multiplanar reconstruction showed cavernous transformation of the portal vein, with the presence of large retroperitoneal collateral vessels communicating with the splenic vein. **c** Direct portography was conducted through percutaneous splenic puncture, confirming the connection between the target collateral vessels and the splenic vein. **d** Under fluoroscopy, use the pig-tail catheter as a guide to target the puncture through the IVC. **e** A stent was successfully placed between retroperitoneal collateral vein and IVC. **f** The spiral-enhanced CT with multiplanar reconstruction images shows that the stent is patent at 1 month after TEPS. *CV* collateral vessels, *PV* portal vein, *SV* Splenic vein, *IVC* inferior vena cava

Based on preoperative imaging evaluation and under ultrasound guidance, a percutaneous transhepatic puncture was carried out on the portal venous tributary within the liver. A 5F/6F catheter sheath was then inserted to explore the PV leading to the superior mesenteric vein (SMV) or splenic vein (SV). If entry to the PV or SMV/SV was not successful via a percutaneous transhepatic route, SV splenic tributary was punctured under ultrasound guidance. Subsequently, the catheter wire was directed to the PV or SMV/SV for portal venography. This was done to further confirm the puncture site of the PV and the shunt pathway as well as to measure the PVP. The PV puncture site, known as the B point, is typically found in the extrahepatic portal vein (EPV), the root of the SMV, the hepatic side of the SV, or within the abdominal or retroperitoneal collateral (Fig. 1). When choosing collateral vessels for shunting, it is essential to consider the collateral' diameter, their blood flow direction in relation to the SMV and SV, their distance and spatial relationship with the inferior vena cava, as well as the surrounding tissues (typically the pancreas, intestines, and major vessels). Additionally, a minimum collateral diameter of 6 mm is deemed suitable for shunting, aiming to establish the safest and most direct shunting pathway. The catheter was superselected into the variceal vascular mass of the esophageal fundus, and the variceal vein was embolized using a mixture of spring coil (Interlock, Boston Scientific, USA) and embolization glue (Beijing Kangpaite Medical Equipment Co., Ltd., China)/papaverine ethyl iodide (Hengri Company, China). Subsequently, a pig-tail catheter or balloon with a diameter of 6–8 mm was positioned at the site of the pseudo-puncture, acting as the marker for the PV system's puncture. In addition, in cases where the hepatic artery and the adjacent portal vein are in close proximity, the use of the hepatic artery catheter as a marker can also be considered. If the patient has previously undergone TIPS, the lower end of the original stent can serve as a marker.

The internal jugular vein of each patient was punctured (the left internal jugular vein could be used if the right side vein was occluded). A RUPS-100 puncture system (Cook, USA) with a 10F-long sheath was then inserted into the inferior vena cava (IVC). After the puncture needle reached the predetermined hepatic vein or IVC (point A), the patient was instructed to hold their breath while adjusting the angle of the puncture needle under fluoroscopy and rotating the C-arm. The C-arm was adjusted to align the puncture point of the hepatic vein/IVC and the marker of the portal venous system with the direction of the puncture needle. Subsequently, the puncture marker was inserted, and the visible shift of the marker catheter or the puncture of the marker balloon indicated a successful puncture. The guide wire and 10F sheath were subsequently placed into the portal venous system to create a working channel. To minimize the risk of major bleeding, balloon dilation of the channel was avoided before the stent was implanted. The VIATORR® stent (Gore, USA) or Fluency™ stent (Bard, USA) was delivered through the 10F-long sheath to completely cover the puncture channel. Subsequently, a 7–8 mm-diameter balloon was employed to expand the puncture channel, with the proximal end of the stent positioned at the junction of the hepatic vein and IVC or within the IVC, while the distal end was placed inside the predetermined portal venous system.

**Fig. 2:**
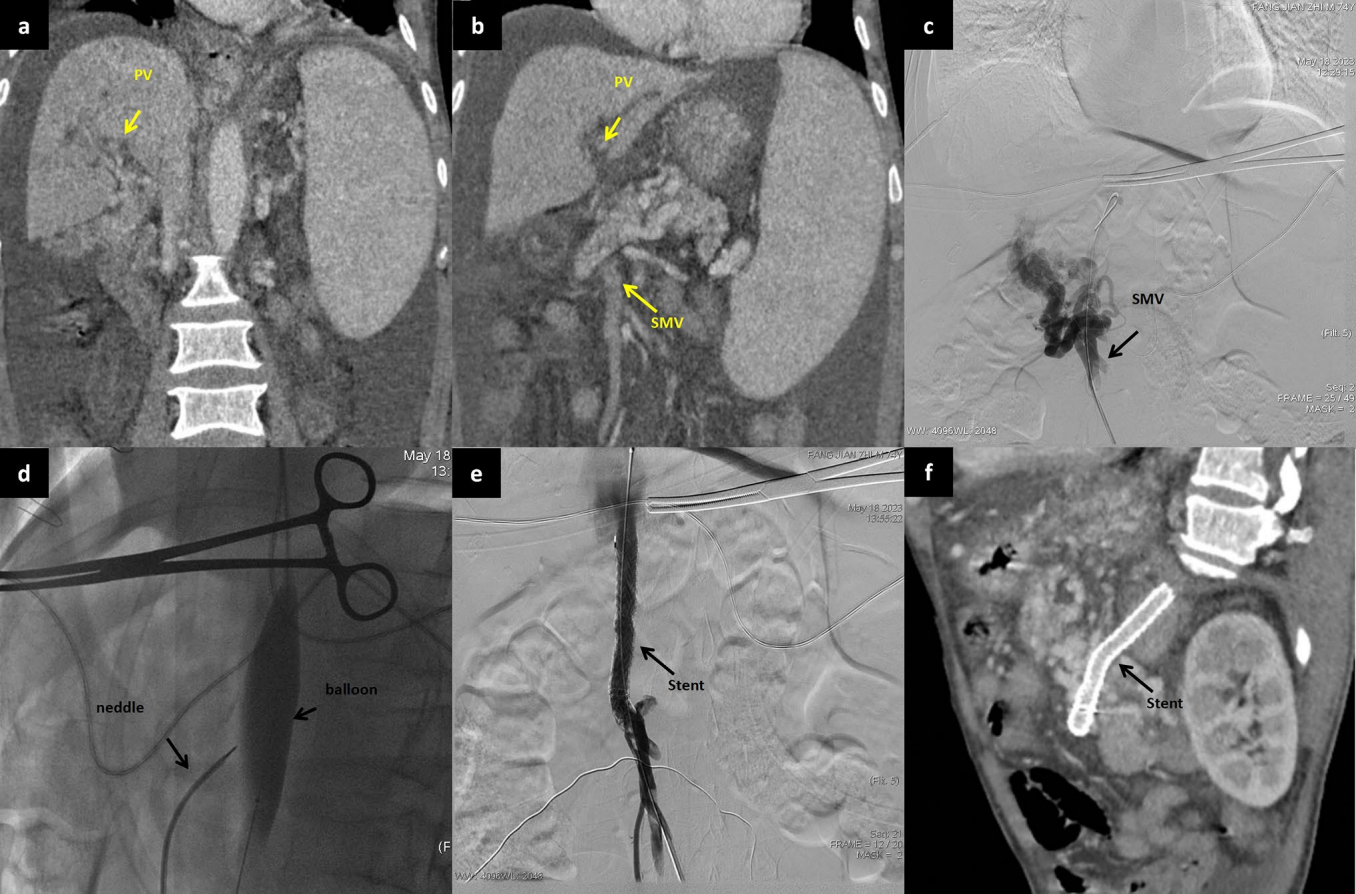
A 74-year-old male patient, who has been dealing with liver cirrhosis for 30 years, has been suffering from recurrent episodes of esophagogastric variceal hemorrhage and recurrent ascites for the recent three years. Previous interventions, including ESVD and LVP, have proven to be ineffective. **a**–**b** The multiplanar reconstruction from the spiral-enhanced CT scans demonstrated cavernous transformation of the portal vein, with patent mesenteric veins. **c** The transabdominal mesenteric venography reveals a patent mesenteric venous system and the presence of extrahepatic collateral vessels. **d** Utilizing a 14 G metal tube, part of the RUPS-100 apparatus, the tube was directed to the proximal region of the SMV with the aid of a guidewire. Following this, a pre-inserted balloon located in the posterior segment of IVC was successfully punctured. **e** A stent was successfully placed between SMV and IVC. **f** The spiral-enhanced CT with multiplanar reconstruction images show that the stent is patent at 1 month after TEPS. *PV* portal vein, *IVC* inferior vena cava, *SMV* Superior mesenteric vein, *ESVD* Endoscopic selective varices devascularization, *LVP* large-volume paracentesis

In cases where the percutaneous transhepatic or percutaneous transsplenic approach proved ineffective, a midline vertical incision was performed below the umbilicus on the abdomen. This was done to expose the tributaries of the SMV with the assistance of surgeons. A 6F catheter sheath was then inserted into the main trunk of the SMV, and a 14 G metal tube and a 5.2F blue catheter, components of the RUPS-100 puncture system, was guided along the wire to the PV trunk or proximal SMV (point B). Under fluoroscopy, a pre-placed balloon in the posterior segment of the IVC was punctured, establishing a working channel in the reverse direction. The stent was subsequently implanted and dilated using the same method to achieve effective portosystemic shunting (Fig. 2).

### Follow-up

Following TEPS, subcutaneous administration of low molecular weight heparin was initiated at a dosage of 4000 IU once every 12 h for 3 days. Additionally, rivaroxaban was administered at a dosage of 10 mg once daily for a minimum of 3 months to prevent shunt dysfunction.

In this study, patients were followed up at 3–7 days, 4–6 weeks, 3 months, 6 months, and 12 months post-procedure. The follow-up included monitoring the duration of postoperative hospitalization, symptoms and signs, shunt patency, and changes in liver and kidney function, coagulation parameters, ammonia, blood leukocytes, hemoglobin, and platelet levels. The patency of the shunt was monitored using color Doppler ultrasound and/or CTA.

## Results

TEPS was successfully performed in 21 out of 22 patients, with their baseline data provided in Table 1. One patient presented with extensive cavernous transformation of the PV, SV, and SMV, resulting in the absence of a suitable puncture pathway from the liver or spleen. Despite the exploration of the SMV during laparotomy, adequate vascular access could not be established, leading to the termination of the surgery. Details, including the age, etiology, clinical manifestation, and treatment history of these 22 patients, are presented in Table 2.

Following TEPS, the portal pressure gradient (PPG) significantly decreased compared to the preoperative levels (24.95 ± 3.19 VS 11.48 ± 1.74, *p* < 0.01). Among them, three patients experienced intraprocedural complications of Grade II–III according to the Clavien–Dindo classification of surgical complications: One patient suffered gallbladder injury (Grade IIIa), and symptoms ameliorated following percutaneous puncture drainage of the gallbladder. The drainage tube was removed 1 month later. Another patient encountered abdominal hemorrhage (Grade II) after percutaneous spleen puncture, and bleeding halted upon sealing the puncture channel with embolization glue. The third patient faced abdominal hemorrhage (Grade II) pre-stent implantation, and bleeding ceased post the deployment of a covered stent. The two patients experiencing intraprocedural bleeding received blood transfusions, and their vital signs remained stable throughout the procedure. No surgery-related fatalities occurred.

**Fig. 3:**
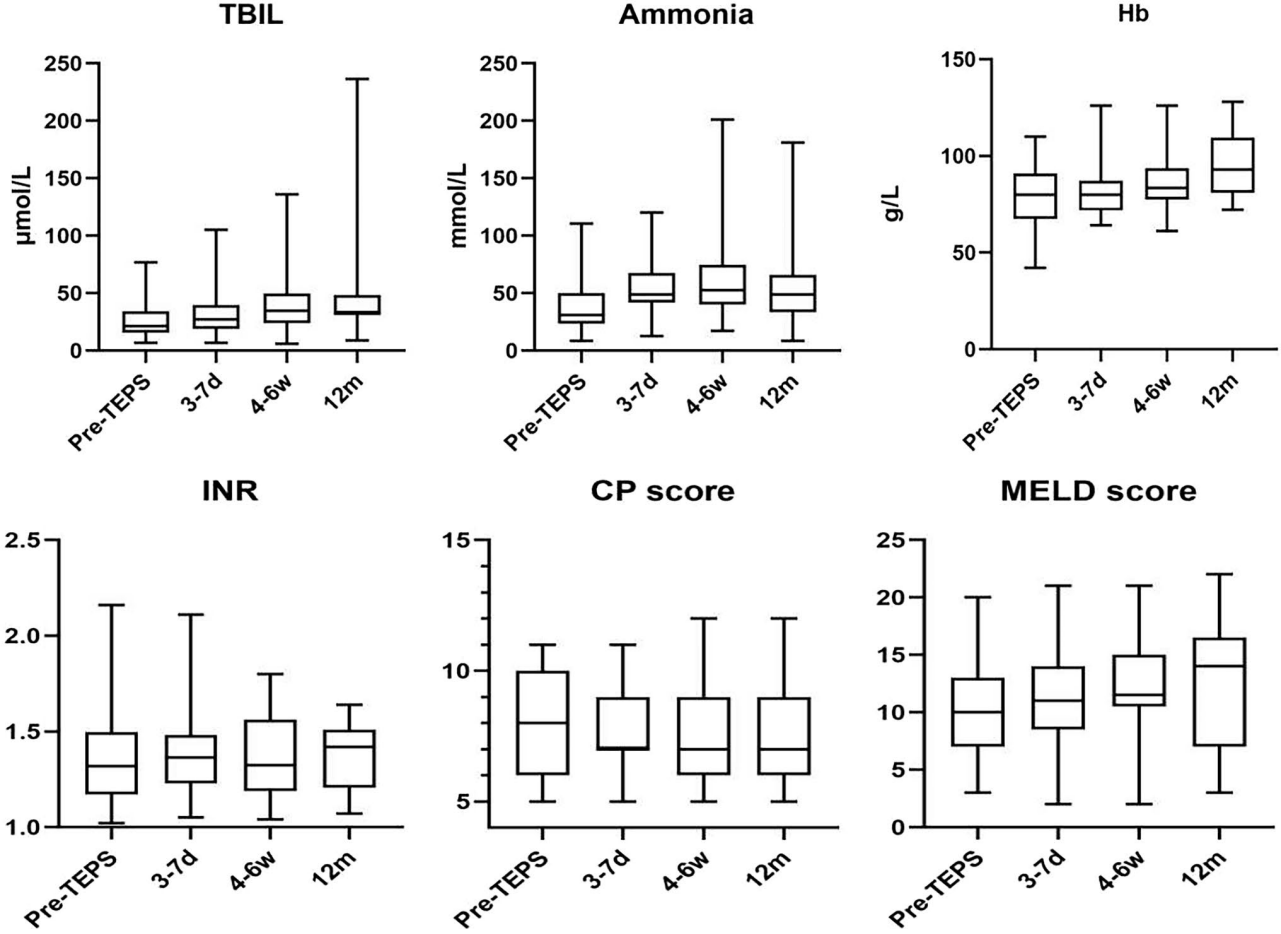
Changes in total bilirubin (TBIL), ammonia, hemoglobin (Hb), international normalized ratio (INR), Child–Pugh (CP) score, and Model for End-Stage Liver Disease (MELD) score were evaluated at various time points. The assessments included measurements before TEPS (*n* = 22), 3–7 days post-TEPS (*n* = 22), and 4–6 weeks post-TEPS (*n* = 22). Furthermore, a subgroup of patients received follow-up at 12 months post-TEPS (*n* = 9)

During a median follow-up of 14 months (range 5–39 months), four patients developed overt HE (West Haven Grading System). They received treatment with medications, such as L-ornithine L-aspartate, lactulose, and rifaximin. Symptoms resolved in one patient after 3 months of TEPS. However, the other three patients continued to experience intermittent grade 2–3 HE. These three patients also developed chronic liver failure. Despite etiological treatment, efforts to eliminate jaundice, promote hepatocyte regeneration, and provide albumin or plasma support, along with necessary artificial liver treatment, were unsuccessful, and these patients succumbed within 7–16 months. Another patient died due to the progression of hepatocellular carcinoma. Shunt dysfunction did not occur in any of the patients during the follow-up. The details of the procedure and outcomes are provided in Table 3, and clinical and biochemical follow-up results are detailed in Fig. 3.

**Table 3:** Details of TEPS procedure and outcomes

No.	Puncture guide method	Route of shunt	Stent Type	Stent Diameter (mm)	Stent Length (mm)	Additional Stent	Post-Dilation Balloon (mm)	PPG pre-TEPS (mmHg)	PPG post-TEPS (mmHg)	Post-TEPS hospitalization duration(days)	Follow-up (months)	Outcome	Intraprocedural complications	delayed complications
1	Percutaneous Transsplenic	EPV-IVC	VTS	8	80		8	27	13	11	39	Survival	Abdominal hemorrhage	None
2	Percutaneous Transsplenic	EPV-HV	VTS	8	70		7	25	14	7	29	Survival	None	None
3	Percutaneous Transsplenic	EPV-IVC	VTS	8	60		8	26	11	7	7	Death due to liver failure	None	CLF/HE
4	Percutaneous Transsplenic	EPV-IVC	VTS	8	80		8	24	13	4	13	Death due to tumor progression	None	None
5	Mark by TIPS stent	EPV-IVC	VTS	8	70		8	29	13	3	29	Survival	None	None
6	Percutaneous Transsplenic	Retroperitoneal collateral-IVC	VTS	8	70		8	29	14	6	9	Death due to liver failure	None	CLF/HE
7	Percutaneous Transsplenic	EPV-IVC	VTS	8	80		7	31	11	7	16	Death due to liver failure	None	CLF/HE
8	Mark by TIPS stent	EPV-IVC	VTS	8	60		8	21	11	3	16	Survival	None	None
9	Transmesenteric	SMV-IVC	VTS	8	80		8	23	10	10	15	Survival	None	None
10	Mark by catheter in hepatic artery	Extrahepatic collateral-IVC	VTS	8	80	8 mm × 8 cm Fluency	8	22	12	16	15	Survival	gallbladder injury	None
11	Mark by catheter in hepatic artery	EPV-IVC	VTS	8	70		8	19	9	6	15	Survival	None	None
12	Percutaneous Transhepatic	EPV-IVC	VTS	8	70		8	28	13	8	14	Survival	None	None
13	Transmesenteric	no succeed	VTS					N/A	N/A	7	14	Survival	None	None
14	Percutaneous Transsplenic	EPV-HV	VTS	8	80		8	22	10	3	14	Survival	None	None
15	Percutaneous Transhepatic	EPV-IVC	VTS	8	80	8 mm × 6 cm Fluency	8	23	11	4	13	Survival	None	None
16	Mark by TIPS stent	EPV-IVC	VCX	8–10	70		8	22	7	5	12	Survival	None	HE
17	Percutaneous Transsplenic	Retroperitoneal collateral-IVC	VCX	8–10	70		8	30	15	10	11	Survival	Abdominal hemorrhage	None
18	Percutaneous Transhepatic	EPV-IVC	VCX	8–10	80		8	18	8	12	9	Survival	None	None
19	Transmesenteric	SMV-IVC	VTS	8	60	10 mm × 5 cm Viabahn	8	27	13	7	9	Survival	None	None
20	Percutaneous Transsplenic	EPV-IVC	VCX	8–10	60		8	29	13	4	8	Survival	None	None
21	Transmesenteric	SMV-IVC	VCX	8–10	70		7	22	9	8	6	Survival	None	None
22	Transmesenteric	SMV-IVC	VCX	8–10	80		8	27	11	10	5	Survival	None	None

*HV* Hepatic vein, *EPV* extrahepatic portal vein, *IVC* inferior vena cava, *SMV* Superior mesenteric vein, *PPG* portal pressure gradient, *CLF* chronic liver failure, *HE* hepatic encephalopathy, *N/A* not applicable or not available, *VTS* VIATORR® TIPS stent grafts, *VCX* VIATORR® Controlled Expansion stent grafts

## Discussion

PVT is a common complication in patients with liver cirrhosis, with an incidence rate of 5–20% [7, 8]. Moreover, the prevalence of PVT is even higher (24–50%) in patients who have undergone splenectomy [9]. However, anticoagulation therapy, endoscopic treatment, or conventional TIPS have demonstrated limited effectiveness in treating chronic PVT and CTPV. Recently, TEPS has been reported as a potential treatment for CTPV, although only a few cases have been documented. To the best of our knowledge, this study is the first to demonstrate the feasibility of precise TEPS using multiple approaches and multiple guidance methods for treating CTPV with portal hypertension.

Due to the low technical success rate and high surgical risks, TIPS is considered a contraindication in CTPV [10, 11]. Various methods have been developed in recent years to enhance the technical success rate in these patients, including the combination of transhepatic or transsplenic puncture guidance[11, 12]. However, despite the utilization of multiple guidance methods, the overall surgical success rate was found to be only 77.8% [4].One important reason is that the traditional methods still require the opening of cavernous transformation of the PV within the liver for shunting. As observed in this study, TEPS demonstrated an impressive success rate of 95.5% (21/22) in the treatment of CTPV. This success can be attributed to two primary factors. First, TEPS provides precise guidance for determining the puncture point and pathway, ensuring accuracy and efficiency during the procedure. Second, TEPS eliminates the need to open the occluded main trunk of the PV, resulting in significant time savings. However, it is essential to note that TEPS is not suitable for all patients with CTPV. Those presenting with occlusion or cavernous transformation of the SMV, SV, or MPV are ineligible for TEPS due to the complexity and challenges associated with their conditions.

Our study indicates that precision TEPS might be a safe option for managing CTPV. However, owing to the limited number of cases, the available data are inadequate to make an accurate assessment of its safety. Data from a larger sample size are necessary for further validation.

During the initial development stages of the TIPS procedure, bleeding emerged as a significant contributor to perioperative mortality in patients[13]. Interventional radiologist have long been concerned about the potential risk of bleeding in extrahepatic portosystemic shunts, as the extrahepatic portal venous system lacks the protection of the Glisson's capsule, unlike the intrahepatic PV [14].In our case series, two patients experienced moderate intra-abdominal bleeding, one during transsplenic puncture and the other during stent deployment. To mitigate the risk of bleeding, we primarily employed three strategies: (1) using ultrasound guidance to puncture the spleen parenchyma, thus avoiding insertion of the puncture needle into the main splenic vein (SV) and employing spring coils or tissue glue to seal the puncture site during sheath removal, and combining this with splenic artery embolization if necessary; (2) minimizing the number of intra-abdominal punctures to precisely guide the placement of the catheter or balloon at the intended location, with the goal of establishing the portal-caval shunt with fewer than two punctures, which represent the fundamental technique ensuring safety in our cases; and (3) refraining from pre-dilation before stent implantation.

In this study, we successfully established a shunt channel between the retroperitoneal collateral and IVC in two patients with only one puncture under precise guidance. However, one of them experienced intra-abdominal bleeding before stent implantation. While reports on lateral branch TIPS are limited, Alexandra Wils documented four cases treated with lateral branch TIPS: two exhibited favorable outcomes, whereas two patients died shortly after the procedure [15]. Tie et al. recently reported on the safety of lateral branch shunting, covering 21 cases primarily focusing on intrahepatic/perihepatic shunts. Whether cases of retroperitoneal collateral vessel shunting were included remains unclear [14]. Based on the aforementioned studies, exploring retroperitoneal collateral shunting appears promising when other suitable shunting pathways are unavailable. However, due to the limited number of cases, the safety of Precision TEPS in these patients cannot be accurately evaluated.

Adjacent organ injury is another crucial risk that requires consideration in Precision TEPS. The pancreas and duodenum, vital organs situated between the portal vein (PV) and inferior vena cava (IVC), should be carefully taken into account when planning the shunt pathway before puncture. Through our analysis of the final shunt pathways in this patients cohort, we observed that the puncture point of the portal venous system (B point) was largely within 2 cm of the extrahepatic portal vein (EPV) trunk, proximal to the superior mesenteric vein (SMV), or proximal to the splenic vein (SV). The A point was predominantly situated in the retrohepatic IVC. Notably, there were no postoperative complications related to organ injury.

Precision TEPS demonstrates a comparable incidence of long-term complications, including hepatic encephalopathy (HE) and chronic liver failure, when compared to conventional TIPS. Earlier studies have indicated an incidence of HE after TIPS ranging from 22.2% to 53.0% [16–19].Within our study, four patients encountered grade II or higher HE (20.0%). Three cases developed chronic liver failure and died due to treatment failure. These patients had similar baseline characteristics of poor liver function, with Child–Pugh scores ranging from 10 to 11 and Model for End-stage Liver Disease (MELD) scores ranging from 13 to 20, indicating that elevated baseline liver function scores continue to be the main contributing factor to the high mortality rate.

The outcomes of Precision TEPS in this case series exhibited favorable shunt efficiency, with no instances of stent occlusion observed at the follow-up endpoint. This could be attributed to the shorter and more direct shunt pathways, along with the favorable anchoring area of the PV. Earlier reports on lateral branch TIPS have indicated that when the PV end of the stent is anchored in small collateral vessels, the shunt efficiency is compromised, resulting in inadequate portal decompression and recurrent bleeding in patients [15, 20]. In contrast to anchoring the PV end within a thrombus or collateral vessels, Precision TEPS anchors it in the SV, the root of the SMV, or larger collateral vessels, resulting in a notable enhancement of shunt efficiency and a reduction in the risk of stent occlusion [14, 15, 20, 21]. However, considering the short follow-up period in this study, additional observations are required to assess its long-term outcomes.

Nevertheless, this study has several limitations. First, Precision TEPS may not be suitable for all patients with CTPV, serving as an alternative when other conventional treatments have proven ineffective. Nevertheless, patients with severe liver and kidney dysfunction may not derive benefits from this procedure. Careful consideration should be given to TEPS when dealing with a complete fibrous transformation of the entire portal venous system without substantial collateral vessels near the IVC or when a safe puncture pathway is unavailable. Notably, in our study, one patient could not undergo successful TEPS due to a sponge-like transformation of the entire portal venous system. Based on our center's experience, approximately 20–30% of patients with CTPV may not be suitable candidates for TEPS. Second, this retrospective study exhibited notable variations in baseline characteristics among patients, encompassing individuals with Budd–Chiari syndrome, liver cancer, or discrepancies in liver function. Consequently, conducting specific performance analyses of Precision TEPS within certain patient subgroups was not feasible. Finally, our study had a limited sample size and a relatively short follow-up period. Larger cohorts and extended follow-up durations are necessary to validate the safety and efficacy of Precision TEPS.

In conclusion, Precision TEPS demonstrates the potential to improve the technical success rate of portosystemic shunts in patients with CTPV while maintaining good safety and efficacy. Our findings suggest that Precision TEPS may offer a safe and effective alternative for CTPV patients unresponsive to conventional treatment.

## Data Availability

Data available on request from the authors.
